# Evidence for Adaptive Introgression of Disease Resistance Genes Among Closely Related *Arabidopsis* Species

**DOI:** 10.1534/g3.117.043984

**Published:** 2017-06-19

**Authors:** Jesper Bechsgaard, Tove Hedegaard Jorgensen, Mikkel Heide Schierup

**Affiliations:** * Department of Bioscience, Aarhus University, 8000 Aarhus C, Denmark; † Bioinformatics Research Center, Aarhus University, 8000 Aarhus C, Denmark

**Keywords:** *Arabidopsis*, introgression, pathogen resistance genes

## Abstract

The generation and maintenance of functional variation in the pathogen defense system of plants is central to the constant evolutionary battle between hosts and parasites. If a species is susceptible to a given pathogen, hybridization and subsequent introgression of a resistance allele from a related species can potentially be an important source of new immunity and is therefore expected to be selected for in a process referred to as adaptive introgression. Here, we survey sequence variation in 10 resistance (*R*-) genes and compare them with 37 reference genes in natural populations of the two closely related and interfertile species: *Arabidopsis lyrata* and *A. halleri*. The *R*-genes are highly polymorphic in both species and show clear signs of *trans*-species polymorphisms. We show that *A. lyrata* and A. *halleri* have had a history of limited introgression for the reference genes. For the *R*-genes, the introgression rate has been significantly higher than for the reference genes, resulting in fewer fixed differences between species and a higher sharing of identical haplotypes. We conclude that *R*-genes likely cross the species boundaries at a higher rate than reference genes and therefore also that some of the increased diversity and *trans*-specific polymorphisms in *R*-genes is due to adaptive introgression.

The genetic systems that underlie resistance to infection and disease in plants and animals often harbor extensive variation in natural populations. Establishing the mechanisms that generate and maintain this variation can further our understanding of how evolution in the wild proceeds and of the evolution of functionally important variation specifically. The common occurrence of long-lived polymorphisms suggests an important role for balancing selection in maintaining variation in pathogen defense genes (Bakker *et al.* 2006; Spurgin and Richardson 2010). However, the relative importance of mechanisms generating variation in pathogen defense genes and particularly the relative importance of hybridization and introgression as a source of adaptive variation is still unresolved.

In plants, the gene-for-gene interaction is one important interface between hosts and their pathogens. Here, resistance to disease is mediated by receptor proteins in the cell membrane or inside the cell that detect pathogen-associated molecules (termed effectors) and initiate defense responses (Jones and Dangl 2006). Receptor proteins are encoded by *R*-genes, of which the most common type encodes a protein with a nucleotide-binding site and a leucine-rich repeat pathogen recognition part (NBS-LRR genes) (Jones and Dangl 2006). Pathogens can overcome the immunity conferred by *R*-genes by altering or deleting the effectors that *R*-proteins detect (Jones and Dangl 2006; Dai *et al.* 2010). Plant *R*-genes often show high sequence or allelic diversity (Bakker *et al.* 2006; Borevitz *et al.* 2007) and some have alleles that transcend species boundaries (Kuang *et al.* 2004; Wang *et al.* 2011; Gos *et al.* 2012; Jouet *et al.* 2015). *Trans*-specific polymorphisms are intriguing because they may either represent ancestral polymorphisms maintained by balancing selection (Charlesworth 2006) or be a consequence of recent gene flow between interfertile species that have resulted in the transfer of advantageous alleles (Klein *et al.* 1998; Hedrick 2013).

Sharing of advantageous alleles due to gene flow is likely if closely related species experience similar environments and are exposed to similar selection pressures (Arnold and Martin 2009). This may be true for plants in relation to disease because closely related plants species are likely to interact with similar or related pathogen species (Barrett *et al.* 2009). In the speciation and divergence of plant hosts, sister species can therefore act as sources of resistance to common pathogens through hybridization and backcrossing (Seehausen 2004). As species diverge, introgression will decrease and shared polymorphisms will become restricted to genomic regions that are not selected against in hybrids, or that carry alleles that are adaptive in the recipient species (Figure 1). It is highly likely that pathogen defense genes are among the last genes in the genome to stop introgressing in this process. Support for this idea comes from the modern human lineage, where a specific haplotype at an HLA-linked locus (Abi-Rached *et al.* 2011) and one at the innate immune gene STAT2 (Mendez *et al.* 2012) were most likely acquired during ancestral admixture with archaic hominins. In plants, a number of functional genes show signs of adaptive introgression [reviewed in Vekemans (2010)] including genes conferring herbivore resistance in *Helianthus* (Whitney *et al.* 2006), resistance to rust fungus in grasses (Jouet *et al.* 2015), and genes involved in the response to viruses in *Arabidopsis* (Novikova *et al.* 2016). Orthologous *R*-genes are common (Guo *et al.* 2011; Hofberger *et al.* 2014) but the extent to which adaptive introgression plays a role in *R*-gene evolution is not yet firmly established.

**Figure 1:**
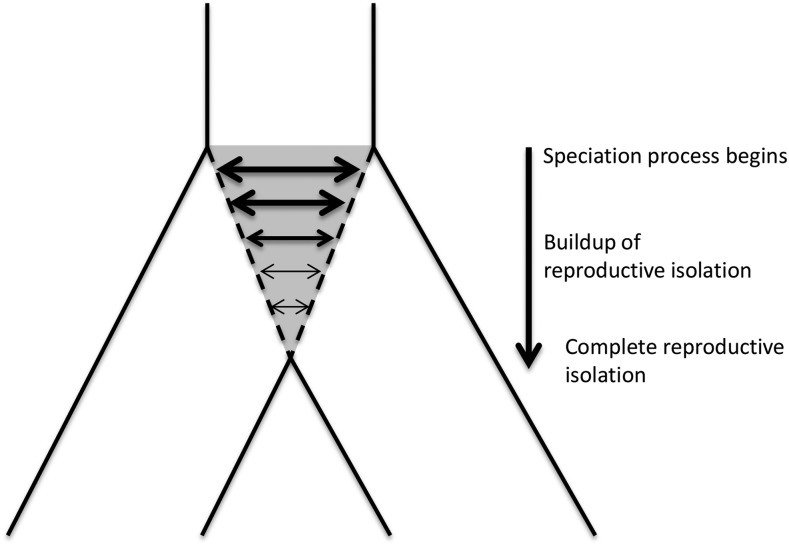
Isolation with migration model of the speciation process of two sister species. The gray area depicts the speciation process, with horizontal arrows indicating introgression. Thinner arrows illustrate the expected decrease in introgression rates during speciation.

The plant genus *Arabidopsis* is relatively young [deepest split 5.5–6 MYA (Hohmann *et al.* 2015; Novikova *et al.* 2016)], with a biogeographic pattern of sympatrically occurring species. Previous studies have provided evidence of frequent hybridization (Sall *et al.* 2003; Schmickl and Koch 2011) and introgression (Ramos-Onsins *et al.* 2004; Novikova *et al.* 2016) between species in the genus. In this study, we focus our attention on the closely related *Arabidopsis lyrata* and *A. halleri*. We expect a role for gene flow in the divergence of these species because they are interfertile and have partly overlapping distributional ranges. Our expectation is supported by a documented fivefold increase in adaptive introgression at a gene controlling pistil self-incompatibility compared to the genomic background (Castric *et al.* 2008). Here, we test the hypothesis that rates of introgression between *A. lyrata* and *A. halleri* are higher in *R*-genes than in other parts of the genome by comparing sequences of 10 *R*-genes and 37 reference genes obtained from several populations, and using the more distantly related *A. thaliana* as an outgroup. We find that several of the *R*-genes show *trans*-specific polymorphisms indicating selective maintenance of variation. Because *R*-gene sequences among species are more similar than expected, we suggest that adaptive introgression is more prevalent at *R*-genes than the rest of the genome.

## Materials and Methods

### Samples

Four or five individuals from each of six *A. lyrata* and four *A. halleri* populations were used. Five of the *A. lyrata* populations belonged to European subsp. *petrea* and cover most of the subspecies distribution (Table 1). One population belonged to American *A. lyrata* sp. *lyrata*. The four *A. halleri* populations were all European.

**Table 1:** Sample locations

Species	Population	GPS Coordinates
*Arabidopsis lyrata*	Iceland*^a^*	64°32N 18°24W
	Germany, Plech	49°39N 11°29E
	Norway, Spiterstulen	61°38N 8°24E
	Sweden, Stubbsand	63°^13^N 18°57E
	Russia, Kärhumäki	62°55N 34°25E
	USA, Indiana	#
*Arabidopsis halleri*	France, Auby	50°24N 03°04E
	France	#
	France	#
	Italy, I9	46°43N 11°25E

GPS, global positioning system; #, exact location is unknown.

a Population 13 in Schierup *et al.* (2008).

### Sequences

Our data consisted of two sets of gene sequences: sequences from 10 *R*-genes and 37 reference genes. The *R*-genes were selected from a list of 127 genes that satisfy two criteria: (a) classified as an *R*-gene in TAIR (Lamesch *et al.* 2012) or in Hofberger *et al.* (2014) and (b) have an ortholog match to *A. lyrata* (Hu *et al.* 2011). We excluded loci that (a) were heterozygous in all individuals and not segregating in a set of offspring from controlled crosses (J. Bechsgaard, unpublished results), or (b) had more than two sequences in any individual. In addition, we used the published *A. lyrata* genome (Hu *et al.* 2011) to assure that the loci included in this study do not have recently duplicated paralogues that could be mistaken as allelic variation. All but one (At1g76950) were NB-LRR genes. We obtained the *R*-gene sequences by PCR and Sanger sequencing (see below), while the reference gene sequences were compiled from three previous publications (Ramos-Onsins *et al.* 2004; Ross-Ibarra *et al.* 2008; Roux *et al.* 2011) and obtained from publicly available databases. Ortholog sequences from *A. thaliana* were downloaded from the 1001 genomes project (Cao *et al.* 2011) to use as an outgroup in analyses. All genes are listed in Supplemental Material, Table S1.

Leaves of *A. halleri* and *A. lyrata* were harvested from juvenile plants and DNA was extracted using a modified version of the CTAB method (Bechsgaard *et al.* 2004). Fragments of 600–800 bp from the *R*-genes were amplified and cloned (see Table S2 for primer information). The PCR protocol in all PCRs was as follows: denaturation at 94° for 3 min followed by 39 cycles of 94° for 30 sec, 52–55° for 60 sec, and 72° for 60 sec, followed by final extension at 72° for 300 sec. At least three clones of each allele were sequenced if two alleles were found and at least eight clones were sequenced if only one allele was found. The cloning primers M13 were used for PCR and sequencing. Two of the genes (At1g52660 and At1g76950) include short introns while the rest include coding sites only.

Table S3 includes the *A. lyrata* gene names that correspond to the gene names of *A. thaliana* and gene positions in the *A. lyrata* genome.

### Sequence analyses

The number of segregating sites (S), number of haplotypes, average pair-wise diversity (π), and estimates of recombination *R*_m_ were obtained in DNAsp v5 (Librado and Rozas 2009) for each species, *R*-gene, and reference locus. The number of fixed and shared polymorphisms between species and polymorphisms unique to each species were estimated using the same software. The divergence between all interspecific pairs of sequences was estimated for synonymous (*K*_s_) and nonsynonymous sites (*K*_n_). Only coding sites were included in the analyses. All π and *K*_s_/*K*_n_ estimates were Jukes–Cantor corrected. For most loci, we assumed that all variation was found. Individuals represented by only one sequence in our analyses were therefore assumed to be homozygous in that locus. Exceptions are the *R*-genes At3g46710 where all 13 sequences sampled represented different alleles, and the reference genes DFR and GS which our data source show not to be exhaustively sampled (Ramos-Onsins *et al.* 2004).

### Phylogenetics

*A. thaliana* sequences were aligned to the sequences from *A. lyrata* and *A. halleri*. Separate phylogenies were constructed in Mega 6.0 (Tamura *et al.* 2013) using the neighbor-joining method with Jukes–Cantor correction, uniform rate among sites, and pairwise deletions. One thousand bootstraps were performed for node support.

### Predicted number of loci with identical sequences

The predicted numbers of identical sequences between species in reference and *R*-genes were simulated using an isolation with migration model in fastsimcoal2 (Excoffier *et al.* 2013) and compared to the observed number of identical sequences in our data. Seven different introgression rates (0, 2 × 10^−8^, 4 × 10^−8^, 4 × 10^−8^, 8 × 10^−8^, 1 × 10^−7^, and 1.5 × 10^−7^ sequences per generation) were used. The rate is defined as the probability of any allele being moved from one species to the other each generation. We used previously published estimates of the species split time, mutation, and recombination rates. No consensus has been reached on split times between *A. lyrata* and *A. halleri* but the most recent estimates are 1.29 MY (Hohmann *et al.* 2015) and 0.56 MY (Novikova *et al.* 2016) leading to 2.58 and 1.12 MY of evolution, respectively. To be conservative, we here assumed 1.12 or 1.5 MY of evolution (0.56 or 0.75 on each branch). In *A. thaliana*, a mutation accumulation experiment yielded a spontaneous mutation rate estimate of 7 × 10^−9^ per site per year (Ossowski *et al.* 2010). We assumed the same mutation rate in *A. lyrata* and *A. halleri*, but adjusted it to 4 × 10^−9^ to match a scenario where genome-wide purifying selection results in a dN/dS value of ∼0.15, as observed in *A. thaliana* (Chen *et al.* 2014). A recent study shows substantial variation in recombination rate within each *R*-gene cluster in *A. thaliana*, varying between 0 and 20 cM/Mb, meaning that some loci within a *R*-gene cluster are recombinationally suppressed while others have high recombination rates (Choi *et al.* 2016). The recombination rate used in our simulations was chosen conservatively to be either 0 or 4.3 × 10^−9^ based on the genome-wide average estimated in *A. thaliana* (Giraut *et al.* 2011). We used two different population sizes of 150,000 and 600,000 for both the ancestral population sizes and current species. For each parameter combination, we simulated (1) 37 loci of length 513 bp to mimic the reference genes and (2) 10 loci of length 730 bp to mimic the resistance loci. Sequences were then sampled in a number corresponding to the average number of sequences in the actual data sets (see Table S1). We counted the number of loci containing identical sequences in the two species from each of 100 replicate simulations for each parameter combination and used boxplots to summarize the results. If the observed numbers of loci with identical sequences in the two species are not included or in the 2.5% tail of simulated distributions, we interpreted the given simulation as significantly different from the observation.

### Data availability

DNA sequences obtained in this study have GenBank accession numbers KY866679–KY867396. Accession numbers for the sequences used for the reference set can be found in Ramos-Onsins *et al.* (2004), Ross-Ibarra *et al.* (2008), and Roux *et al.* (2011).

## Results and Discussion

An average of 48 *A. lyrata* and 23 *A. halleri* sequences for each of the 10 *R*-genes were generated from our 29 *A. lyrata* and 16 *A. halleri* samples (Table S1). None of the loci had more than two alleles per individual, and recent duplications of the investigated genes (which potentially could have inflated diversity) therefore appear unlikely. Species-wide diversity estimates were higher for *R*-genes than for reference genes for both synonymous and nonsynonymous sites (Table 2).

**Table 2:** Estimates of mean nucleotide diversity

	*Arabidopsis lyrata*		*Arabidopsis halleri*	
	Resistance Genes	Reference Genes	Resistance Genes	Reference Genes
π_s_	0.035 (0.025–0.045)	0.026 (0.020–0.032)	0.043 (0.036–0.051)	0.024 (0.018–0.030)
π_a_	0.014 (0.006–0.023)	0.004 (0.003–0.005)	0.008 (0.005–0.011)	0.003 (0.0025–0.005)
π_total_	0.018 (0.011–0.027)	0.010 (0.008–0.012)	0.016 (0.014–0.019)	0.008 (0.006–0.010)
*R*_m_ per site	0.007 (0.004–0.009)	0.004 (0.003–0.006)	0.004 (0.002–0.007)	0.004 (0.003–0.006)

Estimates of mean nucleotide diversity (95% C.I.s) for nonsynonymous (π_s_), synonymous (π_a_), and all sites (π_total_) (Nei and Li 1979). *R*_m_ is the minimum number of recombination events per site (Librado and Rozas 2009).

### Extent of trans-specific polymorphism

If the sequences did not form monophyletic groups in neither *A. lyrata* nor *A. halleri* we considered them to be a *trans*-specific polymorphism. Phylogenetic analyses revealed that sequences from 7 of the 10 resistance loci show sign of *trans*-specific polymorphism of allelic lineages between *A. lyrata* and *A. halleri* (Figure S1). This is a higher fraction than for reference loci (eight of the 37 loci; Fisher’s exact *P* < 0.05). Identical sequences from *A. lyrata* and *A. halleri* were found at 4 of 10 *R*-genes (At1g12220, At1g52660, At4g23440, and At5g47250), and only a single nonsynonymous difference was observed between the most similar sequences in one further gene (At4g26090). Significantly fewer reference genes had identical sequences in the two species (three of the 37 reference genes; GS, At1g62310, and At1g74600; Fisher’s exact, *P* < 0.05). The mean proportion of sites that have fixed differences among the species was 0.0012 in the *R*-genes, whereas the proportion of fixed difference in the reference genes was 0.0070 (5.8 times higher) (permutation test, *P* < 0.05). The mean proportion of sites sharing polymorphisms among species is 0.016 for the *R*-genes, whereas the proportion is 0.0070 for the reference genes (permutation test, 2.3 times lower) (*P* < 0.05). We find no shared polymorphisms with *A. thaliana* in our study, either because *R*-alleles coalesce before the speciation event of *A. thaliana* and the common ancestor of *A. lyrata* and *A. halleri*, or due to a loss of alleles in *A. thaliana*, which probably went through a bottleneck coinciding with its shift from outcrossing to selfing (Bechsgaard *et al.* 2006; Tang *et al.* 2007).

### Predicted number of loci with identical sequences

**Figure 2:**
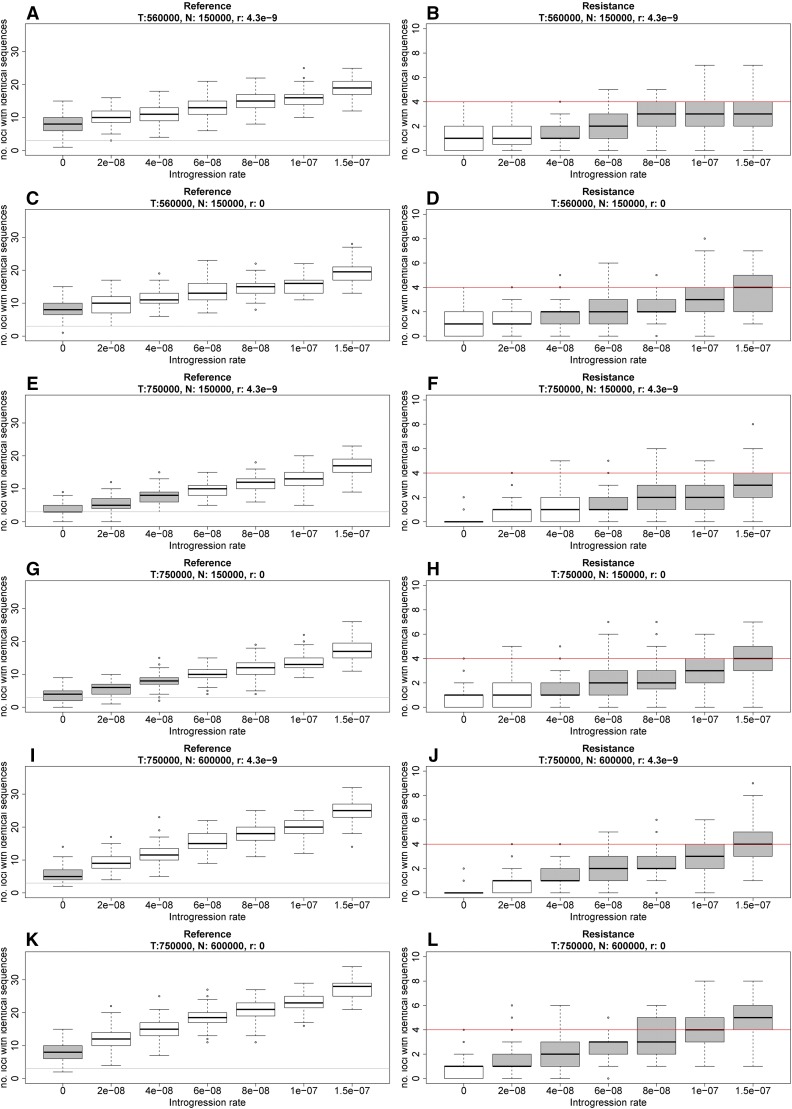
Predicted number (no.) of loci with identical sequences in the two species in reference and resistance genes under different combinations of introgression rates (alleles moved from one species to another/generation), time to species split (*T*), population size (*N*), and recombination rates (*r*) based on coalescent simulations using fastsimcoal2 (Excoffier *et al.* 2013). The mutation rate was 4 × 10^−9^ in all simulations. A, C, E, G, I and K are reference genes, and B, D, F, H, J and L are resistance genes. 100 replicates were simulated for each parameter combination under an “isolation with migration” model. The gray horizontal lines show the number of loci observed with identical sequences in reference genes and the red horizontal lines show the number of loci observed with identical sequences in resistance genes. Box plots in gray represent introgression rates where the observations are not significantly different from the simulations under the given parameter sets.

In our simulations of isolation with migration, introgression between *A. lyrata* and *A. halleri* was not required to explain the observed number of reference loci carrying identical sequences in the two species (Figure 2, left column). Only assuming a split time of 750,000 yr and effective population sizes of 150,000 was the observed number of reference loci with identical sequences also compatible with low introgression rates (Figure 2, E and G).

Previous analyses of the same reference genes do have signatures of introgression consistent with this (Ramos-Onsins *et al.* 2004; Novikova *et al.* 2016). In contrast, introgression is required to explain the observed number of resistance loci with identical sequences in the two species under all sets of parameters (Figure 2, right column). We note that, under one set of parameters (*T*: 750,000 yr, *Ne*: 150,000, and *r*: 0) (Figure 2, G and H), the observed numbers of loci carrying identical sequences in both resistance and reference loci is compatible with an introgression rate of 4e−08. However, we argue that a recombination rate of zero is unrealistic. The *R*-genes have diversities (π) approximately double that of reference genes (0.017 *vs.* 0.008) and their effective population sizes are therefore likely larger than for the reference genes. A larger Ne increased the predicted number of loci with identical sequences slightly (Figure 2, A–H *vs.* and Figure 2, I–L). However, as much as a fourfold difference in Ne between resistance and reference genes (*Ne*: 150000 in reference genes and *Ne*: 600000 in *R*-genes) does not change the overall pattern. We note though that the simulations (*T*: 750,000 and *r*: 4.3e−9) with an introgression rate of 4e−08 are compatible with the observed numbers of loci carrying identical sequences in both resistance and reference loci (Figure 2, E and J).

Changing the combination of parameters in the isolation with migration simulations to explain observations in reference loci would require an even higher introgression rate in order to explain the number of observed resistance loci carrying identical sequences in the two species. We take this as a good indication of higher levels of introgression having occurred in *R*-genes compared to reference genes in *A. lyrata* and *A. halleri*. We note that the two resistance loci At3g46710 and At3g46730 are closely linked in *A. lyrata*, and that they therefore may not segregate independently. This linkage does not cause bias in our results since we observe no identical sequences in the two species in our data.

### Divergence

**Figure 3:**
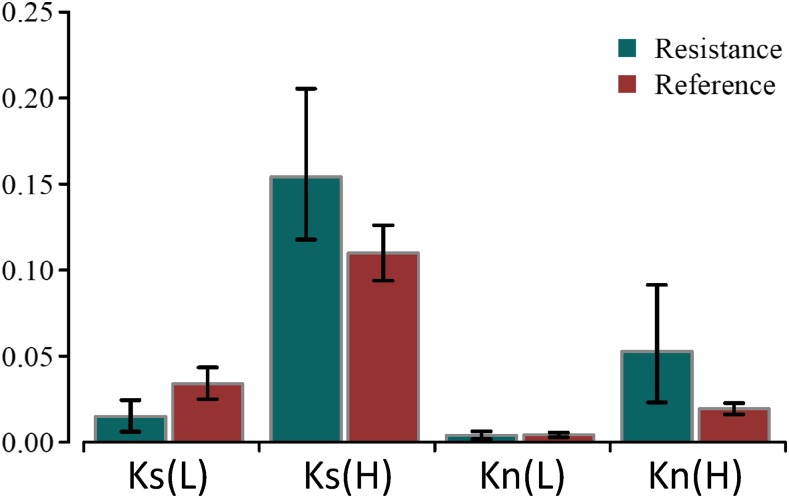
The lowest and highest synonymous [*K*_s_(*L*) and *K*_s_(*H*), respectively] and nonsynonymous [Kn(L) and Kn(H), respectively] divergence of resistance genes and reference genes between *A. lyrata* and *A. halleri* (mean ± 95% C.I.s).

The ranges of divergence values are wider for *R*-genes than for reference genes, with both the lowest synonymous and nonsynonymous divergence [*K*_s_(*L*) and *K*_n_(*L*)] being lower and the highest synonymous and nonsynonymous divergence [*K*_s_(*H*) and *K*_n_(*H*)] being higher (Figure 3). The lowest synonymous divergence of the *R*-genes [*K*_s_(*L*) = 0.0151 (95% C.I. 0.0061–0.0245)] was lower than that of the reference genes [0.0339 (0.0251–0.0435)], but did not differ significantly (*P* = 0.053). The highest synonymous divergence of the *R*-genes [*K*_s_(*H*) = 0.1542 (0.1177–0.2054)] was significantly higher than that of the reference genes [0.1099 (0.0939–0.1260)] (*P* < 0.05). The lowest nonsynonymous divergence of the *R*-genes [*K*_n_(*L*) = 0.0039 (95% CI 0.0019–0.0062)] did not differ from that of the reference genes [0.0043 (0.0029–0.0057)], but the highest synonymous divergence of the *R*-genes [*K*_n_(*H*) = 0.0527 (0.0231–0.0914)] was significantly higher than that of the reference genes [0.0194 (0.0162–0.0227)] (*P* < 0.05).

### Adaptive introgression

Our results show that *R*-genes in *A. lyrata* and *A. halleri* generally have more variation, a larger proportion of variation shared among species, and more identical sequences between species than reference genes. The two species can hybridize and our simulations suggest higher introgression rates at *R*-genes compared to the rest of the genome. This implies some type of selective advantage of introgression of specific resistance types. It also implies that the two species to some extent share a common pool of resistance types and that the variation at these genes within each species is higher than it would be without the opportunity for introgression. Finally, it implies that it is hard to distinguish whether the more widespread *trans*-specific variation at these loci is due to a higher rate of introgression or to balancing selection.

*R*-genes belong to the most polymorphic family of plant genes (Karasov *et al.* 2014a), and with the present study of *A. lyrata* and *A. halleri* we can add to a growing number of *R*-genes that comprise substantial, and often old, within-species variation. At this stage, we can conclude that introgression during species divergence most likely has been important in generating diversity in resistance in *A. lyrata* and *A. halleri* and that the selection imposed on *R*-genes during their divergence appears to have favored a retention of variants already tested by natural selection in sister species. Currently, we can only guess as to how selection acts in our study species. We have empirical evidence from a limited number of host–pathogen systems that pathogens do indeed act to maintain variation locally in populations through fluctuating or frequency-dependent selection [reviewed in Tack *et al.* (2012)]. However, it is clear that the selection regime imposed by pathogens is highly complex because they are dynamic and spatially heterogeneous in their distributions (Tellier and Brown 2007; Moreno-Gamez *et al.* 2013) and form parts of a continuum of more or less specific interactions in a whole community of symbiotic organisms on the host. Most pathogens infect several closely related species and some have host ranges including different taxonomic groups [reviewed in Barrett *et al.* (2009)]. Although the literature shows a bias toward studies of relatively specialized pathogens and their hosts, we now know of several *R*-genes that confer resistance to a wider spectrum of pathogens from the same host (Karasov *et al.* 2014a) or from other host species (Tai *et al.* 1999; Zhao *et al.* 2005; Yang *et al.* 2013). Hosts with broad-spectrum *R*-genes and pathogens with multiple hosts may lead to diffuse selection on disease resistance and contribute to the maintenance of balanced polymorphisms at *R*-genes (Kniskern and Rausher 2007; Karasov *et al.* 2014b). The *R*-genes included in the present study have only partly known functions but at least two genes (*RPS5* and *RPS3/RPM1*) encode proteins in *A. thaliana* that separately recognize effectors from a range of different *Pseudomonas syringae* pathovars (Bisgrove *et al.* 1994; Karasov *et al.* 2014b). As we broaden our view and include more biotic and abiotic components of the host environment, we may be able to more accurately describe the selection regimes imposed on single *R*-genes and hence gain a more comprehensive understanding of the mechanisms generating and maintaining pathogen defense gene diversity in natural populations.

## Supplementary Material

Supplemental material is available online at www.g3journal.org/lookup/suppl/doi:10.1534/g3.117.043984/-/DC1.
