# Supplementary material for: Evidence for Adaptive Introgression of Disease Resistance Genes Among Closely Related Arabidopsis Species
Source: G3 (Bethesda). 2017 Jun 19;7(8):2677–83. doi: 10.1534/g3.117.043984 (PMC5555472; doi:10.1534/g3.117.043984)
Supplement: Supplementary file 1 [file 2677FigureS1.docx]

Figure S1.

Neighbor-joining trees made in Mega 6.0 ([Tamura *et al.* 2013](#_ENREF_48)) of resistance and reference loci analyzed in this study. Node values indicate bootstrap support.

**Resistance genes.**

At1g12220

At1g52660

At1g76950

At2g34930

At3g07040

At3g46710

 At3g46730

At4g23440

At4g26090

At5g47250

**Reference genes.**

At1g01040

At1g03560

At1g04650

At1g06520

At1g06530

At1g10900

At1g10980

At1g11050

At1g15240

At1g59720

At1g62310

At1g62390

At1g62520

 At1g64170

 At1g72390

At1g74600

At2g16870

At2g23170

At2g26140

At2g26730

At2g43680

At2g44900

At2g46550

At3g20820

At3g23590

At3g48690

At3g50740

At3g55060

At3g62890

At3g05630
