# Supplementary material for: Evidence for Adaptive Introgression of Disease Resistance Genes Among Closely Related Arabidopsis Species
Source: G3 (Bethesda). 2017 Jun 19;7(8):2677–83. doi: 10.1534/g3.117.043984 (PMC5555472; doi:10.1534/g3.117.043984)
Supplement: Supplementary file 3 [file 2677TableS2.docx]

Table S2.

| Locus name | Length (bp) | Forward primer | Reverse primer |
| --- | --- | --- | --- |
| Resistance genes |  |  |  |
| At1g12220 | 793 | TACAGGGCGTCAACAAAGGC | GCTTCACCGATAACATTGAGTGC |
| At1g52660 | 702 | CGAGCCACAAGATGGAGAAG | GAGGAAGACCGTCGCACCTGGC |
| At1g76950 | 572 | GTTAAGTTACGAACACAGG | AATCTCTAGTTCCATCGTGGAGG |
| At2g34930 | 729 | CTGAAGTATCTCAACATGGG | CGGATGCTTTTCAAGCTTCTGAG |
| At3g07040 | 793 | TCGAGAGATGTCAGGGTTTGC | AGGAACAACCCTTGTACC |
| At3g46710 | 805 | CCACTGTGTTTGATCTAAGC^#^ | TAGGAGGAGATAGACCTC^#^ |
| At3g46730 | 701 | GTTTACTCCGTCGCCTATTAGAC*,  AATATTACTTAGATCGGTCGTGTC* | CAGAGGACTATGAGATTAATG*,  GCTGATAGATAGAAGCCTAGTC* |
| At4g23440 | 677 | GAAGTTTGGCTACGAGAATGG | TCGTAACATCAGCTTAGCTCG |
| At4g26090 | 783 | GGCGAGTGTACAATTCAGC | GTCCCCTGTTCAGATGCC |
| At5g47250 | 695 | GTGAGATGGCATTGTGGATAG | ACTGGATGATGTGGATGG |
| Reference genes |  |  |  |
| CAD | 957^a^ |  |  |
| CHI | 264 ^a^ |  |  |
| CHS | 1174 ^a^ |  |  |
| DFR | 346 ^a^ |  |  |
| F3H | 450 ^a^ |  |  |
| FAH1 | 1066 ^a^ |  |  |
| GS | 908 ^a^ |  |  |
| MAM-L | 261 ^a^ |  |  |
| At1g01040 | 437 ^b,c^ |  |  |
| At1g03560 | 484 ^b,c^ |  |  |
| At1g04650 | 458 ^b,c^ |  |  |
| At1g06520 | 445 ^b,c^ |  |  |
| At1g06530 | 447 ^b,c^ |  |  |
| At1g10900 | 481 ^b,c^ |  |  |
| At1g10980 | 495 ^b,c^ |  |  |
| At1g11050 | 468 ^b,c^ |  |  |
| At1g15240 | 426 ^b,c^ |  |  |
| At1g59720 | 483 ^b,c^ |  |  |
| At1g62310 | 458 ^b,c^ |  |  |
| At1g62390 | 483 ^b,c^ |  |  |
| At1g62520 | 423 ^b,c^ |  |  |
| At1g64170 | 423 ^b,c^ |  |  |
| At1g72390 | 368 ^b,c^ |  |  |
| At1g74600 | 513 ^b,c^ |  |  |
| At2g16870 | 544 ^b,c^ |  |  |
| At2g23170 | 448 ^b,c^ |  |  |
| At2g26140 | 435 ^b,c^ |  |  |
| At2g26730 | 349 ^b,c^ |  |  |
| At2g43680 | 514 ^b,c^ |  |  |
| At2g44900 | 447 ^b,c^ |  |  |
| At2g46550 | 440 ^b,c^ |  |  |
| At3g20820 | 491 ^b,c^ |  |  |
| At3g23590 | 523 ^b,c^ |  |  |
| At3g48690 | 447 ^b,c^ |  |  |
| At3g50740 | 444 ^b,c^ |  |  |
| At3g55060 | 465 ^b,c^ |  |  |
| At3g62890 | 453 ^b,c^ |  |  |

* forward and reverse primers were used in all combinations.

^#^ does also amplify At3g46530 and At3g46730 sequences.

^a^ from Ramos-Onsins et al (2004)

^b^ from Ross-Ibarra et al (2008)

^c^ from Roux et al (2011)
