# Supplementary material for: Evidence for Adaptive Introgression of Disease Resistance Genes Among Closely Related Arabidopsis Species
Source: G3 (Bethesda). 2017 Jun 19;7(8):2677–83. doi: 10.1534/g3.117.043984 (PMC5555472; doi:10.1534/g3.117.043984)
Supplement: Supplementary file 4 [file 2677TableS3.docx]

Table S3. *Arabidopsis lyrata* gene names from <http://plants.ensembl.org/Arabidopsis_lyrata/Info/Index> - release-33

| *Arabidopsis thaliana* gene names | *Arabidopsis lyrata* gene names,  location of chromosome and position is included |
| --- | --- |
| At1g12220 | fgenesh1_pg.C_scaffold_1001079 cds chromosome:v.1.0:1:4945426:4956021:1 gene:fgenesh1_pg.C_scaffold_1001079 gene_biotype:protein_coding transcript_biotype:protein_coding description:Putative uncharacterized protein [Source:UniProtKB/TrEMBL;Acc:D7KNA4] |
| At1g52660 | fgenesh1_pg.C_scaffold_1003985 cds chromosome:v.1.0:1:28046450:28047772:1 gene:fgenesh1_pg.C_scaffold_1003985 gene_biotype:protein_coding transcript_biotype:protein_coding description:Putative uncharacterized protein [Source:UniProtKB/TrEMBL;Acc:D7KJJ2] |
| At1g76950 | fgenesh2_kg.2__1983__AT1G76950.1 cds chromosome:v.1.0:2:17412762:17417849:1 gene:fgenesh2_kg.2__1983__AT1G76950.1 gene_biotype:protein_coding transcript_biotype:protein_coding gene_symbol:PRAF1 description:Zinc finger protein [Source:UniProtKB/TrEMBL;Acc:D7KTY5] |
| At2g34930 | fgenesh2_kg.4__1518__AT2G34930.1 cds chromosome:v.1.0:4:16607019:16609842:-1 gene:fgenesh2_kg.4__1518__AT2G34930.1 gene_biotype:protein_coding transcript_biotype:protein_coding description:Putative uncharacterized protein [Source:UniProtKB/TrEMBL;Acc:D7LHQ7] |
| At3g07040 | fgenesh2_kg.3__724__AT3G07040.1 cds chromosome:v.1.0:3:2961540:2964400:-1 gene:fgenesh2_kg.3__724__AT3G07040.1 gene_biotype:protein_coding transcript_biotype:protein_coding gene_symbol:RPM1 description:Putative uncharacterized protein [Source:UniProtKB/TrEMBL;Acc:D7L625] |
| At3g46710 | Not present in gene list |
| At3g46730 | fgenesh1_pm.C_scaffold_5000845 cds chromosome:v.1.0:5:12786866:12794517:-1 gene:fgenesh1_pm.C_scaffold_5000845 gene_biotype:protein_coding transcript_biotype:protein_coding description:Putative uncharacterized protein [Source:UniProtKB/TrEMBL;Acc:D7LN43] |
| At4g23440 | fgenesh2_kg.7__1962__AT4G23440.1 cds chromosome:v.1.0:7:8090276:8093333:-1 gene:fgenesh2_kg.7__1962__AT4G23440.1 gene_biotype:protein_coding transcript_biotype:protein_coding description:Transmembrane receptor [Source:UniProtKB/TrEMBL;Acc:D7M947] |
| At4g26090 | scaffold_701696.1 cds chromosome:v.1.0:7:6811069:6813832:-1 gene:scaffold_701696.1 gene_biotype:protein_coding transcript_biotype:protein_coding gene_symbol:RPS2 description:Putative uncharacterized protein [Source:UniProtKB/TrEMBL;Acc:D7MFD1] |
| At5g47250 | scaffold_800067.1 cds chromosome:v.1.0:8:303613:305110:1 gene:scaffold_800067.1 gene_biotype:protein_coding transcript_biotype:protein_coding description:Putative uncharacterized protein [Source:UniProtKB/TrEMBL;Acc:D7MPV2] |
| At1g01040 | fgenesh1_pg.C_scaffold_1000116 cds chromosome:v.1.0:1:511272:518844:-1 gene:fgenesh1_pg.C_scaffold_1000116 gene_biotype:protein_coding transcript_biotype:protein_coding gene_symbol:DCL1 description:Putative uncharacterized protein [Source:UniProtKB/TrEMBL;Acc:D7KQC0] |
| At1g03560 | fgenesh1_pm.C_scaffold_1000233 cds chromosome:v.1.0:1:1053312:1055300:-1 gene:fgenesh1_pm.C_scaffold_1000233 gene_biotype:protein_coding transcript_biotype:protein_coding description:Pentatricopeptide repeat-containing protein [Source:UniProtKB/TrEMBL;Acc:D7KCG2] |
| At1g04650 | Al_scaffold_0001_394 cds chromosome:v.1.0:1:1562090:1566178:-1 gene:Al_scaffold_0001_394 gene_biotype:protein_coding transcript_biotype:protein_coding description:Predicted protein [Source:UniProtKB/TrEMBL;Acc:D7KE64] |
| At1g06520 | scaffold_100650.1 cds chromosome:v.1.0:1:2363049:2365276:-1 gene:scaffold_100650.1 gene_biotype:protein_coding transcript_biotype:protein_coding gene_symbol:ATGPAT1 description:Putative uncharacterized protein [Source:UniProtKB/TrEMBL;Acc:D7KG44] |
| At1g06530 | fgenesh1_pg.C_scaffold_4000007 cds chromosome:v.1.0:4:112345:113575:1 gene:fgenesh1_pg.C_scaffold_4000007 gene_biotype:protein_coding transcript_biotype:protein_coding description:Lipid binding protein [Source:UniProtKB/TrEMBL;Acc:D7LKK7] |
| At1g10900 | fgenesh2_kg.1__1183__AT1G10900.1 cds chromosome:v.1.0:1:4333335:4337333:-1 gene:fgenesh2_kg.1__1183__AT1G10900.1 gene_biotype:protein_coding transcript_biotype:protein_coding description:Phosphatidylinositol-4-phosphate 5-kinase family protein [Source:UniProtKB/TrEMBL;Acc:D7KLK6] |
| At1g10980 | fgenesh2_kg.1__1190__AT1G10980.1 cds chromosome:v.1.0:1:4370070:4372550:-1 gene:fgenesh2_kg.1__1190__AT1G10980.1 gene_biotype:protein_coding transcript_biotype:protein_coding description:Putative uncharacterized protein [Source:UniProtKB/TrEMBL;Acc:D7KLL4] |
| At1g11050 | fgenesh2_kg.1__1193__AT1G11050.1 cds chromosome:v.1.0:1:4384204:4386499:1 gene:fgenesh2_kg.1__1193__AT1G11050.1 gene_biotype:protein_coding transcript_biotype:protein_coding description:Kinase family protein [Source:UniProtKB/TrEMBL;Acc:D7KLL8] |
| At1g15240 | Al_scaffold_0001_1582 cds chromosome:v.1.0:1:6447604:6452044:1 gene:Al_scaffold_0001_1582 gene_biotype:protein_coding transcript_biotype:protein_coding description:Phox-associated domain;Phox-like;Sorting nexin, C-terminal [Source:Projected from Arabidopsis thaliana (AT1G15240) TAIR;Acc:AT1G15240] |
| At1g59720 | fgenesh2_kg.2__473__AT1G59720.1 cds chromosome:v.1.0:2:3770189:3772229:1 gene:fgenesh2_kg.2__473__AT1G59720.1 gene_biotype:protein_coding transcript_biotype:protein_coding gene_symbol:CRR28 description:Putative uncharacterized protein [Source:UniProtKB/TrEMBL;Acc:D7KXS9] |
| At1g62310 | Al_scaffold_0002_290 cds chromosome:v.1.0:2:1822003:1825532:1 gene:Al_scaffold_0002_290 gene_biotype:protein_coding transcript_biotype:protein_coding description:transcription factor jumonji (jmjC) domain-containing protein [Source:Projected from Arabidopsis thaliana (AT1G62310) TAIR;Acc:AT1G62310] |
| At1g62390 | fgenesh2_kg.2__234__AT1G62390.1 cds chromosome:v.1.0:2:1751850:1754685:1 gene:fgenesh2_kg.2__234__AT1G62390.1 gene_biotype:protein_coding transcript_biotype:protein_coding gene_symbol:Phox2 description:Octicosapeptide/Phox/Bem1p domain-containing protein [Source:UniProtKB/TrEMBL;Acc:D7KUR1] |
| At1g62520 | fgenesh2_kg.2__225__AT1G62520.1 cds chromosome:v.1.0:2:1668698:1669793:-1 gene:fgenesh2_kg.2__225__AT1G62520.1 gene_biotype:protein_coding transcript_biotype:protein_coding description:Putative uncharacterized protein [Source:UniProtKB/TrEMBL;Acc:D7KUP7] |
| At1g64170 | fgenesh1_pm.C_scaffold_2000050 cds chromosome:v.1.0:2:424705:427759:1 gene:fgenesh1_pm.C_scaffold_2000050 gene_biotype:protein_coding transcript_biotype:protein_coding gene_symbol:ATCHX16 description:Putative uncharacterized protein [Source:UniProtKB/TrEMBL;Acc:D7KSU8] |
| At1g72390 | fgenesh1_pm.C_scaffold_2001237 cds chromosome:v.1.0:2:15369674:15375358:1 gene:fgenesh1_pm.C_scaffold_2001237 gene_biotype:protein_coding transcript_biotype:protein_coding description:Putative uncharacterized protein [Source:UniProtKB/TrEMBL;Acc:D7KZC9] |
| At1g74600 | fgenesh2_kg.2__1729__AT1G74600.1 cds chromosome:v.1.0:2:16342375:16344528:-1 gene:fgenesh2_kg.2__1729__AT1G74600.1 gene_biotype:protein_coding transcript_biotype:protein_coding description:Putative uncharacterized protein [Source:UniProtKB/TrEMBL;Acc:D7KS35] |
| At2g16870 | fgenesh2_kg.3__3266__AT2G16870.1 cds chromosome:v.1.0:3:21537390:21540965:-1 gene:fgenesh2_kg.3__3266__AT2G16870.1 gene_biotype:protein_coding transcript_biotype:protein_coding description:Putative uncharacterized protein [Source:UniProtKB/TrEMBL;Acc:D7L878] |
| At2g23170 | fgenesh2_kg.4__248__AT2G23170.1 cds chromosome:v.1.0:4:1666339:1668732:-1 gene:fgenesh2_kg.4__248__AT2G23170.1 gene_biotype:protein_coding transcript_biotype:protein_coding gene_symbol:GH3.3 description:GH3.3 [Source:UniProtKB/TrEMBL;Acc:D7LEP8] |
| At2g26140 | fgenesh2_kg.4__529__AT2G26140.1 cds chromosome:v.1.0:4:5781107:5784881:-1 gene:fgenesh2_kg.4__529__AT2G26140.1 gene_biotype:protein_coding transcript_biotype:protein_coding gene_symbol:ftsh4 description:Putative uncharacterized protein [Source:UniProtKB/TrEMBL;Acc:D7LCQ9] |
| At2g26730 | fgenesh2_kg.4__604__AT2G26730.1 cds chromosome:v.1.0:4:10116817:10120026:1 gene:fgenesh2_kg.4__604__AT2G26730.1 gene_biotype:protein_coding transcript_biotype:protein_coding description:Putative uncharacterized protein [Source:UniProtKB/TrEMBL;Acc:D7LES9] |
| At2g43680 | scaffold_403267.1 cds chromosome:v.1.0:4:21338853:21341227:1 gene:scaffold_403267.1 gene_biotype:protein_coding transcript_biotype:protein_coding gene_symbol:IQD14 description:Putative uncharacterized protein [Source:UniProtKB/TrEMBL;Acc:D7LKH0] |
| At2g44900 | scaffold_403428.1 cds chromosome:v.1.0:4:21883519:21887608:-1 gene:scaffold_403428.1 gene_biotype:protein_coding transcript_biotype:protein_coding gene_symbol:ARABIDILLO-1 description:Armadillo/beta-catenin repeat family protein [Source:UniProtKB/TrEMBL;Acc:D7LBT5] |
| At2g46550 | fgenesh2_kg.4__2875__AT2G46550.1 cds chromosome:v.1.0:4:22607095:22609212:-1 gene:fgenesh2_kg.4__2875__AT2G46550.1 gene_biotype:protein_coding transcript_biotype:protein_coding description:Putative uncharacterized protein [Source:UniProtKB/TrEMBL;Acc:D7LEK8] |
| At3g20820 | scaffold_302486.1 cds chromosome:v.1.0:3:9174062:9175206:1 gene:scaffold_302486.1 gene_biotype:protein_coding transcript_biotype:protein_coding description:Leucine-rich repeat family protein [Source:UniProtKB/TrEMBL;Acc:D7KZW8] |
| At3g23590 | fgenesh2_kg.3__2569__AT3G23590.1 cds chromosome:v.1.0:3:10844080:10850295:1 gene:fgenesh2_kg.3__2569__AT3G23590.1 gene_biotype:protein_coding transcript_biotype:protein_coding gene_symbol:RFR1 description:Putative uncharacterized protein [Source:UniProtKB/TrEMBL;Acc:D7L3C9] |
| At3g48690 | fgenesh2_kg.5__1216__AT3G48690.1 cds chromosome:v.1.0:5:13910160:13911289:-1 gene:fgenesh2_kg.5__1216__AT3G48690.1 gene_biotype:protein_coding transcript_biotype:protein_coding gene_symbol:ATCXE12 description:Putative uncharacterized protein [Source:UniProtKB/TrEMBL;Acc:D7LRZ4] |
| At3g50740 | fgenesh2_kg.5__1412__AT3G50740.1 cds chromosome:v.1.0:5:15287713:15289320:-1 gene:fgenesh2_kg.5__1412__AT3G50740.1 gene_biotype:protein_coding transcript_biotype:protein_coding gene_symbol:UGT72E1 description:UDP-glucosyl transferase 72E1 [Source:UniProtKB/TrEMBL;Acc:D7LTI0] |
| At3g55060 | fgenesh2_kg.5__1917__AT3G55060.1 cds chromosome:v.1.0:5:17299602:17303691:-1 gene:fgenesh2_kg.5__1917__AT3G55060.1 gene_biotype:protein_coding transcript_biotype:protein_coding description:Putative uncharacterized protein [Source:UniProtKB/TrEMBL;Acc:D7LV05] |
| At3g62890 | Al_scaffold_0005_3241 cds chromosome:v.1.0:5:20949286:20951090:1 gene:Al_scaffold_0005_3241 gene_biotype:protein_coding transcript_biotype:protein_coding description:Pentatricopeptide repeat (PPR) superfamily protein [Source:Projected from Arabidopsis thaliana (AT3G62890) TAIR;Acc:AT3G62890] |
